# Motor learning principles in the rehabilitation service of speech disorders and Autism

**DOI:** 10.1192/j.eurpsy.2026.11495

**Published:** 2026-07-31

**Authors:** E. Vashdi, R. Popa

**Affiliations:** 1 Yaelcenter, Haifa, Israel; 2 Dinamic Equilibrium, Bucharest, Romania

## Abstract

**Introduction:**

Childhood Apraxia of Speech (CAS) was declared as a motor speech disorder by ASHA (2007). Yet, until then it was mainly addressed as a phonological disorder and until these days, 18 years later, the treatment of CAS is yet to be motor based worldwide. Although the motor declared guidelines by ASHA and by other professionals in other countries, professionals finds it hard to diagnose it clearly due to comorbidity with communication and language disorders. Only very small percentile of children with speech disorder referred to yaelcenter in the last 25 years were diagnosed with CAS although they were all suspected for it.

**Objectives:**

This non clarity might lead to non-accurate treatment since the essence of the syndrome is not addressed. An accurate treatment will integrate knowledge from several domains: communication, Language, Sensory, behavioural, emotional, cognitive and, the most important one for CAS, motor learning.

Motor learning is an area of knowledge which is learnt usually in sport academy, while Its main practical purpose is to improve training methods in sport. The use of motor learning knowledge doesn’t belong to the world of sport primarily but rather to the world of movement wherever it exists. One of the fascinating areas of movement is speech.

**Methods:**

Speech in its basic form is motor based, before it being used as a motor tool for language and communication. It is the most complicated motor task in the human body since for every syllable we activate directly and indirectly over 100 muscles. The timing of all the muscles in producing just one syllable is about 100-200 milliseconds. Full speech utterances which include many sentences, require high motor control due to the high complexity of the motor system. Surprisingly, children acquire speech spontaneously, without direct guiding. It happens because the peripheral and central nervous systems enable that. The children who can’t acquire speech spontaneously due to severe deficit in motor planning, need to practice motor speech tasks repeatedly and accurately.

**Results:**

The VML method (Verbal Motor Learning) is designed to solve this problem. It uses unique speech motor analysis, manual techniques and motor learning principles in order to directly teach the basic milestones of speech for children with CAS. The system is using 20 motor learning principles in depth, being considered in every motor speech task, while bringing high quality and accuracy to treatment. The treatment knowledge is researched based even if not specific to speech.

**Conclusions:**

The presentation will discuss the use of MLP in the service of Apraxia of speech mainly among children diagnosed with Autism where the phenomenon is frequently found.

**Disclosure of Interest:**

None Declared

